# Clinical complete response after trastuzumab deruxtecan 6th-line treatment for postoperative gastric cancer recurrence: a case report

**DOI:** 10.1186/s40792-024-01954-2

**Published:** 2024-06-18

**Authors:** Erika Yamada, Kenichi Iwasaki, Edward Barroga, Toru Sakurai, Masaya Enomoto, Yota Shimoda, Junichi Mazaki, Hiroshi Kuwabara, Akihiro Hoshino, Yutaka Hayashi, Tetsuo Ishizaki, Yuichi Nagakawa

**Affiliations:** 1 Department of Gastrointestinal and Pediatric Surgery, Tokyo Medical University, 6-7-1 Nishishinjuku, Shinjuku-Ku, Tokyo, 160-0023 Japan; 2 Medical English Education Center, Showa University School of Medicine, Tokyo, Japan

**Keywords:** Gastric cancer, Trastuzumab deruxtecan, Complete response, Chemotherapy

## Abstract

**Background:**

Despite the recent developments in the treatment of advanced or recurrent gastric cancer, the median survival time remains shorter than 15 months. Herein, we report a case of postoperative gastric cancer recurrence in which a complete clinical response was achieved with trastuzumab deruxtecan as 6th-line treatment.

**Case presentation:**

A 70-year-old man underwent abdominal contrast-enhanced computed tomography (CT) during follow-up after rectal cancer surgery. The CT revealed an enlarged perigastric lymph node. After further examination, the patient’s condition was diagnosed as gastric cancer cT2N1H0P0M0 cStage IIA. The patient underwent distal gastrectomy and D2 lymph node dissection. The resulting pathological diagnosis was pT1bN3aH0P0 pStageIIB, HER2 score 3+. Abdominal contrast-enhanced CT 19 months postoperatively revealed para-aortic lymph node recurrence, thus systemic chemotherapy courses were planned. The primary treatment was a combination of S-1, cisplatin, and trastuzumab administered in 11 courses. However, there was an enlargement of the para-aortic lymph node which was evaluated as progressive disease. Systematic chemotherapy with various regimens was continued until the 5th-line treatment. However, therapeutic benefits were not achieved and lung metastasis was observed. Trastuzumab deruxtecan (TDXD) was initiated as 6th-line treatment. Abdominal contrast-enhanced CT at 4 months after the start of treatment showed marked shrinkage of the enlarged para-aortic lymph node and disappearance of the lung metastasis in the right upper lung lobe, which was evaluated as partial response (PR). The para-aortic lymph node metastasis was evaluated as PR with only a slight accumulation of SUV-Max 2.66 with a shrinking trend by positron emission tomography-computed tomography (PET-CT) performed after 1 year. Tumor markers CEA, CA19-9, and CA125 also improved significantly. PET-CT after 1 year and 4 months showed no lymph node enlargement or accumulation, indicating a complete response (CR). All tumor markers also normalized. The patient has maintained clinical CR without additional treatment to date.

**Conclusions:**

We report the apparent first case of postoperative gastric cancer recurrence successfully treated with TDXD, achieving clinical CR with TDXD as a 6th-line treatment.

## Background

Trastuzumab deruxtecan (TDXD) is a cytotoxic human epidermal growth factor receptor type 2 (HER2)-directed antibody–drug conjugate approved by several countries worldwide. TDXD is indicated for adult patients with advanced HER2-positive gastric or gastro-oesophageal junction (GOJ) adenocarcinoma with a trastuzumab-based regime treatment history. The phase II DESTINY-Gastric01 trial [1] showed significant effective outcomes of intravenous TDXD compared with standard chemotherapy (physician’s choice of intravenous paclitaxel or irinotecan), improving the overall survival of Asian adults with advanced HER2-positive gastric or GOJ adenocarcinoma who had a history of receiving 2 or more previous therapies. Moreover, in the phase II DESTINY-Gastric02 trial [2], TDXD induced a stable response in US or European patients with unresectable or metastatic HER2-positive gastric or GOJ adenocarcinoma.

HER2 overexpression is amplified in approximately 10%–15% of gastric cancer [3, 4], a predictor of recurrence and poor prognosis. Treatment of HER2-positive gastric cancer involves several options including surgery, chemotherapy, and other systemic therapies. Among the various options, trastuzumab plus chemotherapy is the standard treatment for patients with unresectable HER2-positive gastric or GOJ adenocarcinoma. Recently, several studies have provided evidence that TDXD is an effective treatment option in previously treated patients with advanced HER2-positive gastric or GOJ adenocarcinoma [1, 5]. Therefore, the addition of TDXD as a treatment option may improve the treatment outcomes of patients with HER2-positive gastric cancer.

Herein, we report an extremely rare case of postoperative gastric cancer recurrence in a patient who received TDXD as a 6th-line treatment, which achieved a clinical CR even without follow-up treatment.

## Case presentation

**Fig. 1:**
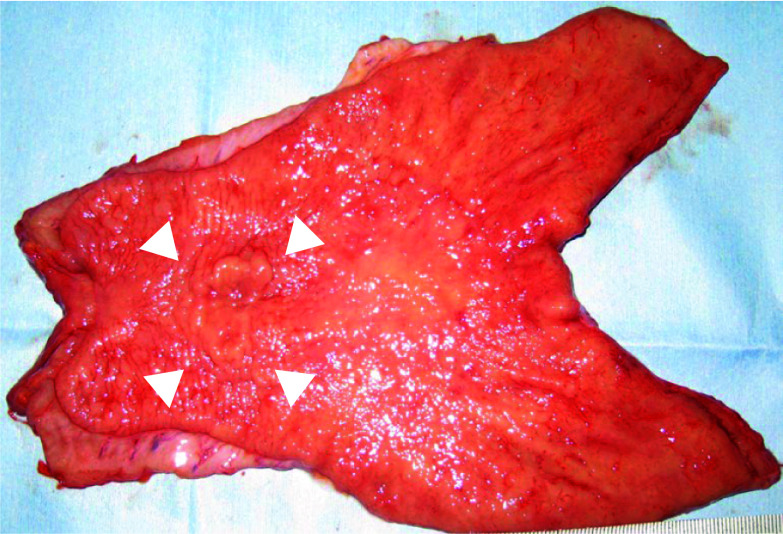
Gross findings of the resected stomach. A 30 × 20 mm type0-IIc tumor was observed in the posterior wall of the middle stomach (white triangles)

**Fig. 2:**
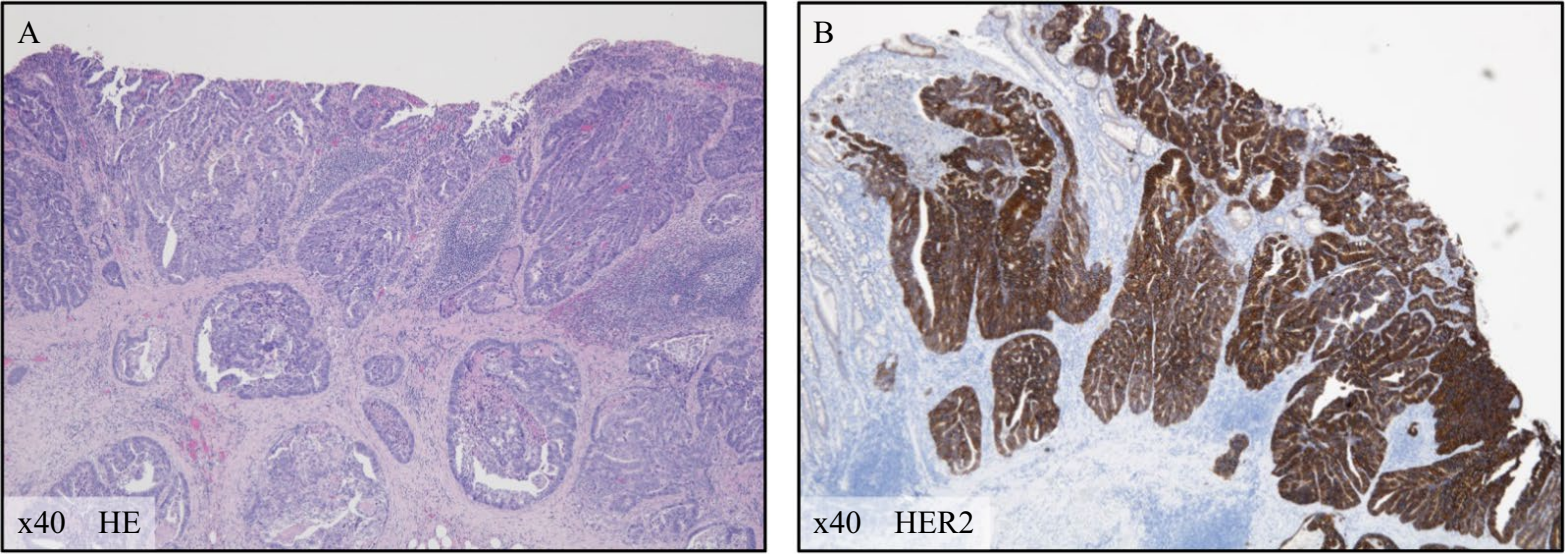
Pathological findings of the resected sample. **A** Hematoxylin and eosin staining of the specimen confirmed well-differentiated tubular adenocarcinoma cells infiltrating the submucosal layer. **B** Human epidermal growth factor receptor type 2 (HER2) immunohistochemical staining of the specimen confirmed a strong expression of HER2 protein in the tumor cells

**Fig. 3:**
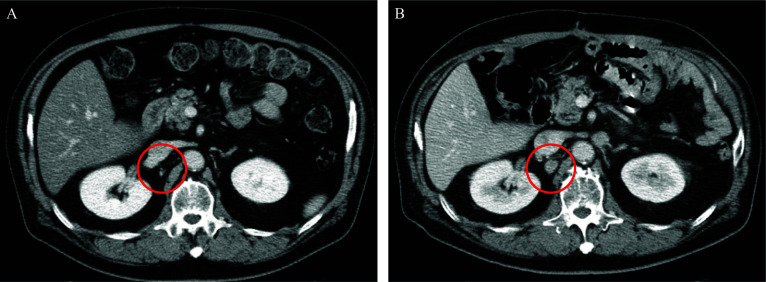
A regular follow-up contrast-enhanced computed tomography (CT) scan revealed a para-aortic lymph node recurrence (**B**) which was not detectable before gastrectomy (**A**) (red circle)

A 70-year-old man without any particular chief complaint underwent contrast-enhanced computed tomography (CT) as a 4-year and 6-month follow-up examination of the laparoscopic low anterior resection for rectal cancer. The CT revealed a significant enlargement of the lymph node around his stomach. Further examination by upper gastrointestinal endoscopy showed a 0-IIc lesion in the posterior wall of the gastric body. The pathological diagnosis by biopsy was a well-differentiated adenocarcinoma. Further examinations specifically revealed gastric cancer cT2N1H0P0M0 cStage IIA according to the Japanese Classification of Gastric Carcinoma (15th edition) [6]. The patient underwent curative distal gastrectomy with D2 lymph node dissection. The pathological diagnosis was adenocarcinoma (tub2 > tub1), M-Post, 30 × 20 mm, type0-IIc, pT1bN3aH0P0 pStageIIB, HER2 score 3+ (Figs. 1; 2A, B). The intraoperative and postoperative courses were good without any complications, and the patient was discharged on postoperative day 13. Although starting adjuvant chemotherapy was suggested, the patient declined to receive additional treatment because of the adverse events he experienced with adjuvant chemotherapy after rectal cancer surgery. Thus, regular follow-up by blood sampling and imaging tests was planned. One year and 7 months after surgery, a regular follow-up contrast-enhanced CT scan revealed a para-aortic lymph node recurrence which was not detectable before gastrectomy (Fig. 3A, B).

Chemotherapy was administered with S-1 plus cisplatin and trastuzumab as the primary treatment based on the pathological diagnosis of HER2-positive adenocarcinoma. The size of the lymph node was stable without showing any shrinkage, and after administering 11 courses for approximately 8 months, contrast-enhanced CT of the abdomen showed increased para-aortic lymph node metastasis, which was evaluated as progressive disease (PD) based on the *response evaluation criteria in solid tumors* (RECIST) version 1.1. As a second-line chemotherapy, a combination of ramucirumab and paclitaxel was used for 6 courses for approximately 6 months. However, this combination was ineffective and the condition was judged as PD because of the enlarged para-aortic lymph node revealed by contrast-enhanced CT. Further spread of the para-aortic lymph node metastases and an adverse event of Grade 3 general malaise based on the *common terminology criteria for adverse events* (CTCAE) version 5.0 were observed after treatment with irinotecan plus cisplatin as a 3rd-line chemotherapy for 9 courses for approximately 6 months. Nivolumab was selected as a 4th-line regimen and was administered for 37 courses for approximately 1 year and 6 months, followed by contrast-enhanced CT which revealed a marked enlargement of the para-aortic lymph node evaluated as PD based on the RECIST version 1.1. As a 5th-line chemotherapy, capecitabine plus oxaliplatin was selected and administered. After 13 courses for approximately 11 months, contrast-enhanced CT showed an increase in the para-aortic lymph node metastasis and a new nodular shadow in the upper lobe of the right lung, which led to a diagnosis of pulmonary metastasis. At this stage, 4 years and 2 months have passed since the chemotherapy for the recurrence has been started.

**Fig. 4:**
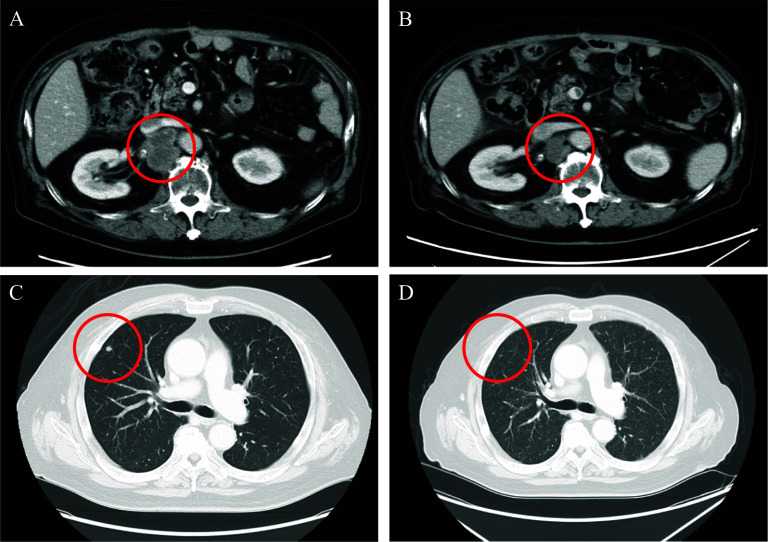
Contrast-enhanced CT findings after chemotherapy. **A**, **B** Prominent shrinkage of the enlarged metastatic para-aortic lymph node (red circle). **C**, **D** Disappearance of the lung metastases in the right upper lung lobe (red circle)

**Fig. 5:**
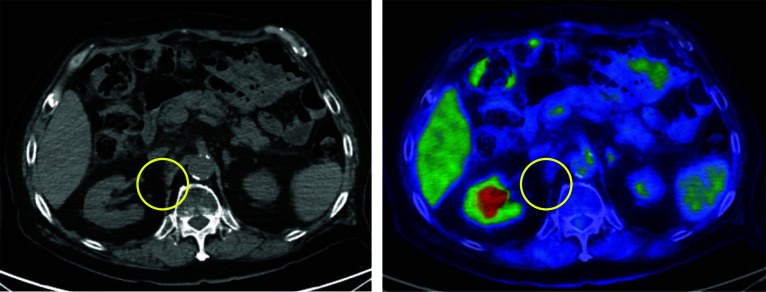
Positron emission tomography-computed tomography findings after chemotherapy. There was no evidence of tumor recurrence, and the condition was diagnosed as clinical complete response (yellow circle)

**Fig. 6:**
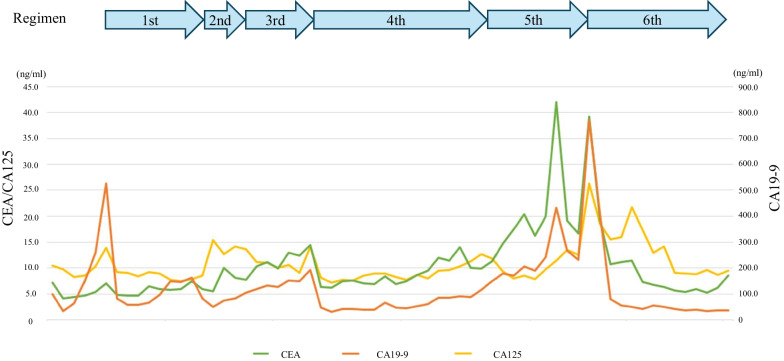
Temporal progression of the therapeutic course and transition of the tumor markers

During this time period, TDXD was covered by insurance for HER2-positive gastric or GOJ adenocarcinoma, with a history of receiving 2 or more previous therapies since 2020, TDXD was selected as 6th-line treatment. Contrast-enhanced CT was performed for evaluation after completion of the 5th course (approximately 4 months after the start) and revealed prominent shrinkage of the enlarged metastatic para-aortic lymph node (Fig. 4A, B) and disappearance of the lung metastases in the right upper lung lobe (Fig. 4C, D), with an evaluation of partial response (PR) based on the RECIST version 1.1. Positron emission tomography-computed tomography (PET-CT), which was performed after completion of the 12th TDXD course for approximately 1 year after the start, showed that the para-aortic lymph node metastasis had further decreased in size with only a slight accumulation of the maximum standardized uptake value (SUV-Max) as 2.66. The evaluation was PR based on the RECIST version 1.1. Tumor markers also showed significant improvement compared with the values at the start of the 6th-course of treatment. The levels of both carcinoembryonic antigen (CEA) and cancer antigen 19-9 (CA19-9) showed a reduction within the normal limit. After 15 TDXD courses for approximately 1 year and 4 months after the start of the 6th treatment course, evaluation by contrast-enhanced CT and PET-CT scan showed no evidence of tumor recurrence indicating a clinical complete response (CR) (Fig. 5). At this time, approximately 6 years has passed since the detection of the postoperative recurrence, and nearly 2 years from the start of the 6th-line chemotherapy. As of this writing, the patient has maintained clinical CR without the need for any further treatment. Additional administration of TDXD has not been performed after achieving clinical CR, and the patient has undergone regular outpatient follow-up using tumor markers and contrast-enhanced CT. The temporal progression of the therapeutic course and the transition of the tumor markers are shown in Fig. 6.

## Discussion

To our knowledge, this is the first case of postoperative gastric cancer recurrence achieving a clinical CR after a 6th-line TDXD treatment. We searched case reports in PubMed using the terms “gastric cancer”, “trastuzumab deruxtecan”, and “complete response”, with an unlimited publication record. However, we found no articles reporting on the achievement of CR of postoperative gastric cancer recurrence after TDXD treatment. The importance of our present case report is that it contributes evidence on achieving effective treatment of postoperative gastric cancer recurrence.

In recent years, various clinical trials have demonstrated the efficacy of cytotoxic anticancer agents, molecular targeted agents, and immune checkpoint inhibitors, leading to the expansion of systematic chemotherapy options for unresectable advanced or recurrent gastric cancer. As a result, a small number of patients with long-term survival have been observed [7, 8]. Despite the recent development in treatments, the median survival time (MST) for advanced or recurrent gastric cancer remains shorter than 15 months [7, 9, 10].

According to the ToGA trial, HER2-positive gastric cancer accounts for 22.1% of all gastric cancers. The study compared standard chemotherapy alone, which consisted of a combination of capecitabine or fluorouracil (5-FU) with cisplatin, with chemotherapy combined with trastuzumab for primary treatment of HER2-positive advanced gastric cancer and oesophagogastric junction cancer. The MST was significantly extended from 11.1 months in the chemotherapy-alone group to 13.8 months in the trastuzumab-combined group [4].

The chemotherapy regimens recommended in the 6th edition of the Japanese guidelines for the treatment of gastric cancer, which was revised in July 2021 [9], are described differently for HER2-positive and HER2-negative cases. For HER2-positive advanced or recurrent gastric cancer cases, cisplatin or oxaliplatin plus trastuzumab in combination with capecitabine or S-1 is recommended as the primary therapy. Although there are no HER2-positive-specific treatment options for second-line treatment, the combination of paclitaxel and ramucirumab is encouraged based on the RAINBOW trial results [9, 10]. Additionally, the WJOG7112G study has rejected the significance of continuing trastuzumab as a second-line therapy after the usage of trastuzumab-containing combination therapy as 1st-line treatment for HER2-positive gastric cancer. [9, 11]. For a 3rd-line therapy, TDXD is recommended [9].

TDXD is an antibody–drug conjugate targeting HER2. It was approved in September 2020 for HER2-positive inoperable advanced or recurrent gastric cancer that progressed after chemotherapy. The antibody component is a humanized immunoglobulin G1 (IgG1) monoclonal antibody with the same amino acid sequence as that of trastuzumab, and the drug component utilizes a derivative of the topoisomerase I inhibitor, irinotecan. During DNA replication in cancer cells, there is a process where topoisomerase I cuts 1 strand of the DNA to temporarily relieve the DNA’s helical structure. Inhibiting topoisomerase I can induce cells to undergo apoptosis [12].

The present patient did not experience any adverse events of TDXD, including interstitial pneumonia which requires particular attention because of its severity. The incidence rate of interstitial pneumonia associated with TDXD administration for gastric cancer is 9.6% and often occurs within 1 year after treatment initiation [13]. Prompt consultation with a respiratory specialist is necessary when respiratory symptoms occur, particularly as early symptoms may resemble the cold symptoms, and regular imaging follow-up is recommended.

In the present case, after introducing TDXD as a 6th-line treatment, the patient achieved clinical CR for 2 years which has been maintained without additional treatment or TDXD-related adverse events. According to the DESTINY-Gastric01 trial, a CR rate of 9.2% (11 out of 119 cases) was reported [1], and patients who showed a response in at least 1 imaging test were evaluated as responders. Therefore, the fact that a long-term CR was achieved in this case is considered highly significant. The reason for introducing TDXD at such a late phase (i.e., 6th-line treatment) rather than according to guidelines was because it had not been locally approved until September 2020. If the transition to 3rd-line treatment in this case had occurred after the approval of TDXD in September 2020, TDXD could have been introduced at the 3rd-line stage, potentially reducing chemotherapy duration, decreasing the number of regimens, shortening the period during which patients experience symptoms and psychological stress owing to cancer, preventing adverse events that may occur through the use of other regimens, and reducing the medical costs. The reason why TDXD was exclusively effective and that trastuzumab could not achieve clinical CR even though they both specifically target HER-2 positive cases is unclear. However, the effectiveness of TDXD is primarily suggested to be due to its payload component. It is implied that the payload part played a critical role in the effectiveness of TDXD, even in cases such as in the present patient when trastuzumab is ineffective. Additionally, Ogitani et al. have indicated that TDXD is absorbed by cancer cells, demonstrating its antitumor effects. Furthermore, it penetrates adjacent tumor cells owing to its high membrane permeability, suggesting that it may offer a significant therapeutic benefit, the so-called bystander killing effect [14]. It is possible that these characteristics of TDXD contributed to its therapeutic effects that could not be achieved with trastuzumab.

Regarding the order of usage of immune checkpoint inhibitors including nivolumab and TDXD, the present case used nivolumab before TDXD administration. However, the optimal third or later line treatment remains unclear for HER-2-positive gastric cancer patients [15]. According to the ATTRACTION-2 trial, the long-time survival from nivolumab treatment was unsatisfactory, which suggests that the majority of gastric cancer patients may not benefit from nivolumab monotherapy [16]. Matsumoto et al. reported that the pre-administration of immune checkpoint inhibitors maybe the key to achieving a favorable prognosis and enhancing the efficacy of TDXD [17]. In the present case, nivolumab was administrated prior to TDXD and was able to achieve a stable disease control for more than 1 year and 6 months. Regarding the order of use, there is still much room for discussion, but from our experience of the present case, using both nivolumab and TDXD can potentially contribute to long-term survival.

Perhaps the most insightful aspect of the present case is that this is apparently the first case report of achieving clinical CR for postoperative gastric cancer recurrence with the use of TDXD. Our case report suggests that there may be some potential for the treatment of postoperative gastric cancer recurrence with a poor prognosis. Although careful attention is required for adverse events such as interstitial pneumonia, it is suggested that the proactive use of TDXD may be beneficial for patients with HER2-positive gastric cancer in the future.

## Conclusion

To our knowledge, this is the first case report of clinical CR after TDXD as a 6th-line treatment for postoperative gastric cancer recurrence. The response of the current patient indicates prolonged survival of HER-2 positive patients with recurrence after gastric cancer surgery. Additionally, TDXD showed good safety and tolerability, suggesting its potential use for the successful treatment of HER2-positive recurrent gastric cancer. Accumulation of cases and additional investigations including large-scale, multicenter clinical studies, are required to definitively clarify the efficacy expansion and indications of TDXD.

## Data Availability

The datasets generated or analyzed during the current study are available from the corresponding author on reasonable request.

## Abbreviations

TDXD: Trastuzumab deruxtecan
HER2: Human epidermal growth factor receptor type 2
GOJ: Gastro-oesophageal junction
CT: Computed tomography
PD: Progressive disease
RECIST: Response evaluation criteria in solid tumors
CTCAE: Common terminology criteria for adverse events
PR: Partial response
PET-CT: Positron emission tomography-computed tomography scan
SUV-Max: Maximum standardized uptake value
CEA: Carcinoembryonic antigen
CA19-9: Cancer antigen 19–9
